# Perceptions of Quality of Care Among Users of a Web-Based Patient Portal: Cross-sectional Survey Analysis

**DOI:** 10.2196/39973

**Published:** 2022-11-17

**Authors:** Rachael Lear, Lisa Freise, Matthew Kybert, Ara Darzi, Ana Luisa Neves, Erik K Mayer

**Affiliations:** 1 National Institute for Health and Care Research Imperial Patient Safety Translational Research Centre Institute of Global Health Innovation Imperial College London London United Kingdom; 2 Imperial College Healthcare NHS Trust London United Kingdom

**Keywords:** electronic health records, personal health records, patient participation, patient safety, care quality, digital health literacy

## Abstract

**Background:**

Web-based patient portals enable patients access to, and interaction with, their personal electronic health records. However, little is known about the impact of patient portals on quality of care. Users of patient portals can contribute important insights toward addressing this knowledge gap.

**Objective:**

We aimed to describe perceived changes in the quality of care among users of a web-based patient portal and to identify the characteristics of patients who perceive the greatest benefit of portal use.

**Methods:**

A cross-sectional web-based survey study was conducted to understand patients’ experiences with the Care Information Exchange (CIE) portal. Patient sociodemographic data were collected, including age, sex, ethnicity, educational level, health status, geographic location, motivation to self-manage, and digital health literacy (measured by the eHealth Literacy Scale). Patients with experience using CIE, who specified both age and sex, were included in these analyses. Relevant survey items (closed-ended questions) were mapped to the Institute of Medicine’s 6 domains of quality of care. Users’ responses were examined to understand their perceptions of how portal use has changed the overall quality of their care, different aspects of care related to the 6 domains of care quality, and patient’s satisfaction with care. Multinomial logistic regression analyses were performed to identify patient characteristics associated with perceived improvements in overall care quality and greater satisfaction with care.

**Results:**

Of 445 CIE users, 38.7% (n=172) reported that the overall quality of their care was better; 3.2% (n=14) said their care was worse. In the patient centeredness domain, 61.2% (273/445) of patients felt more in control of their health care, and 53.9% (240/445) felt able to play a greater role in decision-making. Regarding timeliness, 40.2% (179/445) of patients reported they could access appointments, diagnoses, and treatment more quickly. Approximately 30% of CIE users reported better care related to the domains of effectiveness (123/445, 27.6%), safety (138/445, 31%), and efficiency (174/445, 28.6%). Regarding equity, patients self-reporting higher digital health literacy (odds ratio 2.40, 95% CI 1.07-5.42; *P*=.03) and those belonging to ethnic minority groups (odds ratio 2.27, 95% CI 1.26-3.73; *P*<.005) were more likely to perceive improvements in care quality. Across ethnic groups, Asian and British Asian patients perceived the greatest benefits. Increased frequency of CIE use also predicted perceived better care quality and greater satisfaction with care.

**Conclusions:**

A large proportion of CIE users perceived better care quality and greater satisfaction with care, although many portal users reported no change. The most favorable perceived improvements related to the domain of patient centeredness. With national policy directed toward addressing health disparities, patient portals could be valuable in improving care quality for ethnic minority groups. Future research should test the causal relationship between patient portal use and care quality.

## Introduction

### Background

Web-based patient portals are thought to contribute to improvements in care quality by providing patients with access to their personal health information, empowering them to self-manage their health and become true partners in their own care [1]. As the trend toward patients being able to access their electronic health records accelerates [2], there is a pressing need to evaluate the impact of patient portals, understand their risks and benefits from both patient and provider perspectives, and generate evidence to inform future health policy [3].

Although care is traditionally delivered through face-to-face clinical consultations, patient-provider communication through patient portals is increasingly common [1]. The Care Information Exchange (CIE) is the largest shared personal health records program in the United Kingdom and provides patients with secure web-based access to their health and social care records. Patients can additionally use CIE in different ways: for example, to self-monitor their health by linking home health care devices (eg, activity tracker and blood pressure monitor) to the portal, to communicate with care providers through messaging and videoconferencing, and to check appointments and test results and be signposted to useful weblinks and resources by health and care professionals.

One of the most influential guides for evaluating health care initiatives is the Institute of Medicine’s framework, which includes 6 domains of quality of care: effectiveness, safety, timeliness, efficiency, patient centeredness, and equity [4,5]. Effectiveness is about achieving optimal health outcomes by providing appropriate treatment to patients who could benefit and avoiding the underuse and misuse of health services [4,5]. Patient safety seeks to prevent patients from being harmed by the care that is intended to help them [4,5]. Timeliness is about reducing harmful waits and delays, whereas efficiency is about minimizing resource waste [4,5]. Patient centeredness respects patient preferences and needs and values and ensures these are incorporated into clinical decision-making [4,5]. Equity ensures that care does not vary in quality because of differences in patient characteristics such as ethnicity or geographic location [4,5].

Over the last decade, a considerable body of evidence has uncovered important barriers to portal use, enabling the development of portals in line with patient and health service need [6-8]. In contrast, relatively few studies have investigated the relationship between patient portals and quality of care. Some prior evidence demonstrates the beneficial effects of patient portal use, particularly in supporting preventive behaviors and disease control in people with chronic conditions [3,7,9]. A number of studies have documented positive associations between patient portals and patient safety [3,7,10-13], including improved adherence to medical regimens and reductions in medication discrepancies [3]. However, evidence for the impact of patient portals across other domains of quality is sparse, and where evidence does exist, findings have been mixed [3,7]. Among patients who use web-based portals, little is known about which sociodemographic groups perceive the greatest benefits of access to their personal health records. Furthermore, policy makers agree that more evidence is needed to understand the impact of tools that use digital technologies amidst concerns over a growing *digital divide* [14].

### Objectives

The aims of this study were to describe perceptions of quality of care among users of a web-based patient portal and to identify the characteristics of portal users who perceive the greatest benefit of portal use.

## Methods

### Study Design, Participants, and Data Collection

A cross-sectional survey study was conducted to explore patients’ views and experiences of using CIE. The questionnaire was administered via Qualtrics (web-based survey platform) and was open for completion between July 1, 2018, and July 1, 2019. At the time of the survey, CIE was deployed to the diverse 2.3 million patients treated in North West London, including patients residing in London and in other geographic locations across England. CIE held records from hospitals and general practitioners in North West London and from 15 hospitals outside of London, in Birmingham, Bristol, Liverpool, Manchester, Scotland, and Wales. All patients registered with the CIE at the time of the survey were invited via email to complete the questionnaire (n=27,411). The email explained the purpose of the study; informed consent was obtained. Patients accessed the questionnaire via a web link in the portal. Patients had to be aged at least 18 years to be registered with CIE. Not all patients registered with CIE were using the portal. With this data set, we have previously evaluated differences between users and nonusers of CIE with respect to their sociodemographic characteristics and demonstrated the importance of addressing educational aspects and digital literacy to ensure equitable and sustainable portal adoption [15]. Our further work has sought to evaluate the impact of web-based patient portals on safety and quality of care from the patient’s perspective. Our recent study found that a large proportion of patients are able and willing to use patient portals to participate in identifying and rectifying errors in their personal health records [16]. This study builds on previous work to understand patients’ perceptions of the impacts of CIE across 6 domains of care quality.

**Figure 1:**
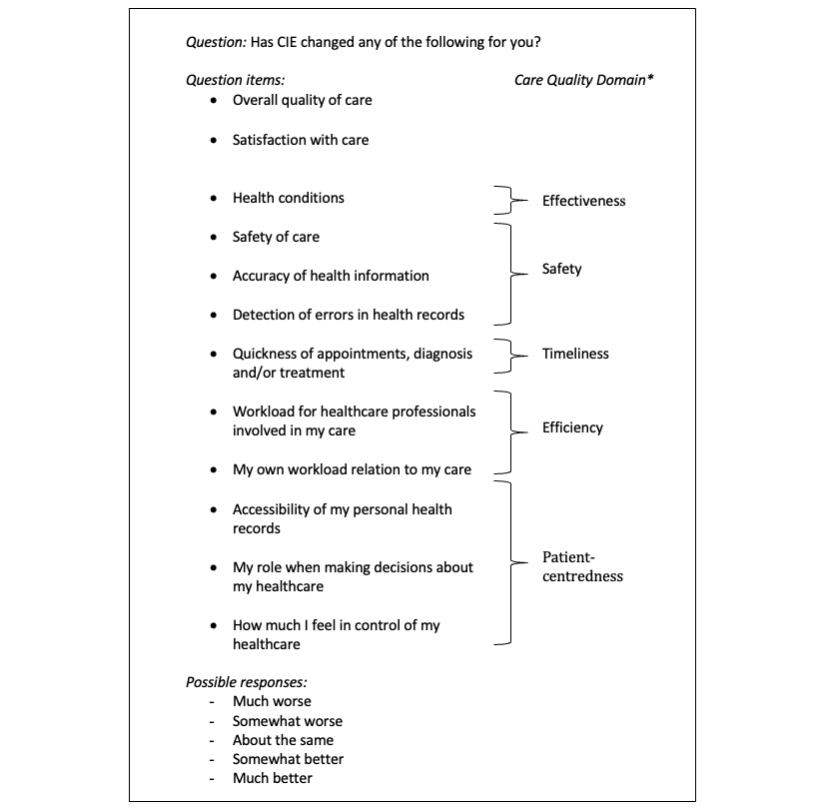
Questionnaire items mapped to care quality domains. The domain of equity was assessed using the methods described in this section. *As defined by the Institute of Medicine, 2001 [5]. CIE: Care Information Exchange.

For these analyses, we included patients who had previously accessed and used the CIE portal. We excluded patients who did not provide basic demographics regarding age and sex. Considering this population, a CI of 95%, and a margin of error of 5%, the minimum sample size to ensure representativeness was calculated as 379 respondents. We mapped relevant survey items to the Institute of Medicine’s domains of quality of care: effectiveness, safety, timeliness, efficiency, and patient centeredness [5,17]. Patients’ responses to 12 multiple-choice, closed-ended question items were analyzed. Figure 1 outlines the 12 question items, with mapping to care quality domains.

To evaluate equity, we conducted multivariable regression analyses to determine associations between patients’ sociodemographic characteristics and perceptions of the impact of CIE on overall care quality and satisfaction with care. The following information was collected to input into multivariable analyses: age, sex, ethnicity, native language, education level, digital health literacy, motivation to be involved in own care, and health status. Respondents’ level of motivation to be involved in their own care was assessed via a multiple-choice question (“In general, how motivated to be involved in your healthcare are you?” Possible responses: “A little,” “A moderate amount,” “A lot,” and “Very much”). Digital health literacy was assessed using the eHealth Literacy Scale (eHEALS), developed and validated by Norman and Skinner [18]. The eHEALS tool is an 8-item measure of patients’ combined knowledge, comfort, and perceived skills in finding, evaluating, and using internet health resources for health problems [18]. The 8 items are answered on a 5-point Likert scale (1, strongly disagree to 5, strongly agree); total eHEALS scores range from 8 to 40, with a higher score indicating higher digital literacy.

### Data Analysis

We used descriptive statistics to summarize respondent characteristics and patients’ responses to question items. Counts and proportions were calculated for categorical variables; means and SDs were calculated for continuous variables. Age was categorized into bands (<30, 31-40, 41-50, 51-65, and ≥65 years). Owing to the small numbers of patients self-identifying to individual categories of ethnic minority background, ethnicity was categorized as “ethnic minorities” or “White.”

We conducted multinomial regression analyses to identify sociodemographic characteristics that predict patient-perceived improvement in overall care quality and greater satisfaction with care. To overcome the issue of sparse counts in multivariable modeling (Tables S1 and S2 in Multimedia Appendix 1), “age,” “motivation to be involved in own care,” “digital health literacy,” and “frequency of CIE use” were treated as dichotomous variables, and respondents reporting sex as “other” were excluded. Consistent with previous studies, we selected an eHEALS score ≥26 to indicate higher digital health literacy and <26 to indicate lower digital health literacy [19-23]. We also combined categories of the dependent variable (ie, “much worse” and “somewhat worse” were analyzed as a single category; equally, “somewhat better” and “much better” were combined into 1 category). We performed univariate multinomial logistic analyses to identify possible predictors to include in the multivariable model. We adopted the approach by Hosmer et al [24,25] for variable selection: (1) variables that demonstrated significance (*P*<.25) in the univariate analyses were entered into the preliminary multivariable model; (2) variables that were nonsignificant at *P*>.05 according to the likelihood ratio test were removed one at a time according to the variable with the highest *P* value (backward elimination); (3) to check for suppressor effects, variables excluded during backward selection were re-entered separately into the regression model (forward selection). Only variables that were significant at *P*<.05 (Likelihood Ratio Test) were retained in the final multinomial regression models. Model quality comparisons were conducted using the Akaike Information Criterion [26], and goodness-of-fit was assessed using the Pearson chi-square statistic [25]. Effect estimates are presented as odds ratios (ORs) with their 95% CIs.

To assess the effects of excluding patients with missing data regarding age and sex, we compared the sociodemographic characteristics of the missing data sample (n=78) and the analysis sample (n=445). We ran a Pearson chi-square test of homogeneity (*χ*^2^) to compare the distribution of item responses between the analysis sample and the missing data sample for the perceived impact of CIE on the overall quality of care and satisfaction with care.

Analyses were conducted using Microsoft Excel (version 16.54) and SPSS software (version 27; IBM Corp).

### Ethics Approval

The study was approved as a Service Evaluation at Imperial College Health care NHS Trust (registration number: 296/2018).

### Reporting

We followed the reporting recommendations in the Strengthening the Reporting of Observational Studies in Epidemiology Statement (Multimedia Appendix 2). [27].

## Results

### Respondent Characteristics

Of 1083 patients who responded to the survey, 523 (48.29%) patients who were “CIE users” completed the questionnaire. CIE users who provided basic demographic details regarding age and sex were included in the analysis (445/523, 85.1%; +117% of the minimum target sample size); 14.9% (78/523) of respondents with missing data for age and sex were excluded.

Of 445 respondents, most (n=313, 70.3%) were aged >50 years and 276 (62%) were female. Approximately 1 in 5 (97/445, 21.8%) respondents belonged to an ethnic minority group. Most (292/445, 65.6%) respondents were educated to the degree level or higher, and the mean eHEALS score was 33.6 (SD 6.4, range 8-40); a score ≥26 indicates higher digital health literacy. Of 445 patients, 177 (39.8%) patients reported being in good health; 162 (36.4%) of patients reported that the status of their health was poor. Most (278/445, 62.5%) patients reported being very motivated in their own care. Most (284/445, 63.8%) patients said they used CIE at least once a month, and 93.2% (415/445) of patients said they found CIE useful. Patient characteristics are presented in Table 1.

**Table 1:** Respondent characteristics (N=445).

		Respondents
**Sex, n (%)**		
	Male	167 (37.5)
	Female	276 (62)
	Other	2 (0.4)
	No response	N/A^a^
**Age group (years), n (%)**		
	<30	22 (4.9)
	31-40	48 (10.8)
	41-50	62 (13.9)
	51-64	166 (37.3)
	>65	147 (33)
	No response	N/A
**Ethnicity, n (%)**		
	Asian or British Asian	44 (9.9)
	Black African or Black Caribbean or Black British	20 (4.5)
	Mixed or multiple ethnic groups	11 (2.5)
	Other	22 (4.9)
	White	343 (77.1)
	No response	5 (1.1)
**Geographic location, n (%)**		
	London	284 (63.8)
	Other locations in England	145 (32.6)
	No response	16 (3.6)
**Education, n (%)**		
	Secondary school or below	118 (26.5)
	Undergraduate or professional degree	180 (40.4)
	Postgraduate or higher	112 (25.2)
	No response	35 (7.9)
**Language, n (%)**		
	English	379 (85.2)
	Non-English	58 (13.0)
	No response	8 (1.8)
eHealth literacy (eHEALS^b^ score), mean (SD; range)		33.6 (6.4; 8-40)
**Overall health status, n (%)**		
	Good or very good	177 (39.8)
	Neither good nor poor	106 (23.8)
	Poor or very poor	162 (36.4)
	No response	0 (0)
**Motivation to be involved in own care, n (%)**		
	Not very much	6 (1.3)
	A moderate amount	43 (9.7)
	A lot	116 (26.1)
	Very much	278 (62.5)
	No response	2 (0.4)

^a^N/A: not applicable.

^b^eHEALS: eHealth Literacy Scale.

### Patients’ Perceptions of the Impact of CIE on the Overall Quality of Care

Patients were asked to consider how CIE has changed the overall quality of care they receive. Of 429 patients who answered this question, 172 (38.7%) reported that the quality of their care was better with CIE. A further 54.6% (243/445) said that their care was about the same, and 3.2% (14/445) of patients said their care was worse (Multimedia Appendix 3).

### Patients’ Perceptions of the Impact of CIE on Satisfaction With Care

When asked to consider how CIE has changed and how satisfied they are with their care, 43.6% (194/445) of patients said their care was better, 47.6% (212/445) said their care was the same, and 4.3% (19/445) said their care was worse. In addition, 4.5% (20/445) of patients did not respond to this question (Multimedia Appendix 3).

### Patients’ Perceptions of the Impact of CIE Across 6 Domains of Care Quality

#### Overview

Patients’ responses to a further 10 survey items revealed their perceptions of how CIE use has changed the care they receive across the following domains of quality of care: effectiveness, safety, timeliness, efficiency, and patient centeredness (Table 2).

**Table 2:** Survey items and patients’ responses, mapped to the Institute of Medicine’s domains of health care quality (N=445).

Health care quality domain^a^ and survey item: “Has CIE changed any of the following...?”			Missing data, n (%)		Much worse, n (%)		Somewhat worse, n (%)			About the same, n (%)		Somewhat better, n (%)		Much better, n (%)
**Effective**														
	Health conditions	30 (6.7)		7 (1.6)		9 (2)			276 (62)		61 (13.7)		62 (13.9)	
**Safe**														
	Safety of care	33 (7.4)		7 (1.6)		7 (1.6)			260 (58.4)		68 (15.3)		70 (15.7)	
	Accuracy of health information	25 (5.6)		9 (2)		11 (2.5)			187 (42.0)		117 (26.3)		96 (21.6)	
	Detection of errors in health records	32 (7.2)		8 (1.8)		13 (2.9)			246 (55.3)		70 (15.7)		76 (17.1)	
**Timely**														
	Quickness of appointments, diagnosis, and/or treatment	29 (6.5)		13 (2.9)		12 (2.7)			212 (47.6)		77 (17.3)		102 (22.9)	
**Efficient**														
	Workload for health care professionals involved in my care	31 (7.0)		7 (1.6)		15 (3.4)			265 (59.6)		63 (14.2)		64 (14.4)	
	My own workload relating to my care	28 (6.3)		11 (2.5)		23 (5.2)			209 (47)		92 (20.7)		82 (18.4)	
**Patient centeredness**														
	Accessibility of my personal health records	16 (3.6)		6 (1.3)		9 (2.0)			72 (16.2)		112 (25.2)		230 (51.7)	
	My role when making decisions about my health care	6 (1.3)		11 (2.5)^b^		11 (2.5)^b^			188 (42.2)^c^		240 (53.9)^d^		240 (53.9)^d^	
	How much I feel in control of my health care	6 (1.3)		19 (4.3)^e^				19 (4.3)^e^		147 (33)^c^		273 (61.2)^f^		273 (61.2)^f^

^a^As defined by the Institute of Medicine [5].

^b^I feel I have less of a role.

^c^No change.

^d^I feel I have more of a role.

^e^I feel I have less control.

^f^I feel I have more control.

#### Effectiveness

Patients were asked whether CIE use had changed their health condition. Most (276/445, 62%) patients responded that their health condition was about the same; however, 27.6% (123/445) patients reported that their health condition had improved with CIE use. Only 3.6% (16/445) said their health condition was worse.

#### Safety

Although many (260/445, 58.4%) patients reported that the safety of the care was the same with CIE; 31% (138/445) felt that their care was safer. Approximately half (213/445, 47.9%) believed that CIE had led to improvements in the accuracy of their health information, and 32.8% (146/445) of patients felt CIE was associated with better detection of errors in the health record. Only 3.2% (14/445) of patients felt the safety of their care was worse with CIE.

#### Timeliness

Approximately 40% (179/445) of patients felt that the timeliness of their care (being able to access appointments, diagnoses, and treatment quickly) had improved with CIE. Only 5.6% (25/445) said the timeliness of their care was worse, and 47.6% (212/445) said the timeliness of their care was about the same.

#### Efficiency

Patients were asked whether CIE had changed the workload relating to their health, including both patients’ own workload and the workload of health professionals involved in their care. Many (209/445, 47%) patients reported that their own workload was about the same; however, 28.6% (174/445) felt that their workload was better, and 7.7% (34/445) felt their workload was worse. Regarding the impact of CIE on the workload of health professionals, 39.1% (174/445) of patients perceived that this had improved, 59.6% (265/445) believed it to be about the same, and 5% (22/445) thought that it was worse.

#### Patient Centeredness

Most (342/445, 76.9%) patients reported that CIE had improved the accessibility of their personal health records. A few (72/445, 16.2%) patients felt that the accessibility of their records was about the same with CIE, whereas only 3.3% (15/445) said their records were less accessible. More than half (240/445, 53.9%) of the survey respondents reported that CIE had led to them having more of a role in decision-making, and 61.3% (273/445) feel they have more control of their health care. Only 2.5% (11/445) of patients reported feeling they have less of a role, and 4.3% (19/445) felt they have less control of their health care with CIE.

#### Equity

To identify the characteristics of CIE users who perceived better overall quality of care and greater satisfaction with care with portal use, patient characteristics and survey responses were entered into univariate and multivariable multinomial regression models.

For the survey item, “How has CIE changed the overall quality of care you have received?” the final multivariable multinomial regression model with 3 predictor variables (ethnicity, digital health literacy, and frequency of CIE use) predicted significantly better than the null (intercept) model (*P*<.001) and Pearson chi-square statistic indicated satisfactory model fit (*χ*^2^_8_=14.4; *P*=.07). The results of the regression are presented in Table 3. Patients with higher digital health literacy (eHEALS score≥26) were more likely to report that the overall quality of their care was better with CIE use (OR 2.40, 95% CI 1.07-5.42; *P*=.03). Compared with their White counterparts, patients self-identifying to an ethnic minority group were also more likely to perceive improvements in care quality based on CIE use (OR 2.27, 95% CI 1.26-3.73; *P*=.005). Across ethnic groups, 68% (30/44) of Asian and British Asian patients reported better overall quality of care with CIE use, compared with 45% (9/20) of Black or African or Caribbean or Black British patients, 36.6% (120/328; missing data, n=15) of White patients, and 36% (4/11) of patients from mixed or multiple ethnic groups (Table S1 in Multimedia Appendix 4).

**Table 3:** Multinomial regression results of patient characteristics and perceived change in overall quality of care with Care Information Exchange use.

		Univariate^a^						Multivariable^a^				
		Worse care quality vs about the same			Better care quality vs about the same			Worse care quality vs about the same			Better care quality vs about the same	
		Odds ratio (95% CI)	*P* value	Odds ratio (95% CI)		*P* value	Odds ratio (95% CI)		*P* value	Odds ratio (95% CI)		*P* value
**Sex**												
	Female	Reference	Reference	Reference		Reference	Reference		Reference	Reference		Reference
	Male	0.47 (0.13-1.74)	.26	1.26 (0.84-1.88)		.26	N/A^b^		N/A	N/A		N/A
**Age (years)**												
	≥65	Reference	Reference	Reference		Reference	Reference		Reference	Reference		Reference
	≤64	1.35 (0.41-4.42)	.63	1.28 (0.84-1.94)		.26	N/A		N/A	N/A		N/A
**Ethnicity**												
	White	Reference	Reference	Reference		Reference	Reference		Reference	Reference		Reference
	Ethnic minority	1.88 (0.49-7.18)	.36	2.27 (1.37-3.78)		.002	2.44 (0.61-9.80)		.21	2.27 (1.26-3.73)		.005
**Native language**												
	English	Reference	Reference	Reference		Reference	Reference		Reference	Reference		Reference
	Non-English	2.56 (0.66-9.91)	.18	1.81 (1.02-3.21)		.04	—^c^		—	—		—
**Education**												
	Secondary or below	Reference	Reference	Reference		Reference	Reference		Reference	Reference		Reference
	Undergraduate or professional	4.60 (0.55-38.23)	.16	0.85 (0.53-1.38)		.51	—		—	—		—
	Postgraduate or higher	4.00 (0.44-36.76)	.22	0.73 (0.42-1.25)		.25	—		—	—		—
**Digital literacy**												
	Lower digital health literacy	Reference	Reference	Reference		Reference	Reference		Reference	Reference		Reference
	Higher digital health literacy	1.57 (0.20-12.63)	.67	2.51 (1.15-5.45)		.02	1.51 (0.18-12.42)		.70	2.40 (1.07-5.42)		.03
**Health status**												
	Neither good nor poor	Reference	Reference	Reference		Reference	Reference		Reference	Reference		Reference
	Poor	0.72 (0.20-2.60)	.62	1.29 (0.77-2.16)		.34	N/A		N/A	N/A		N/A
	Good	0.52 (0.14-2.02)	.35	1.22 (0.73-2.03)		.45	N/A		N/A	N/A		N/A
**Motivation to be involved in own care**												
	Not very much or a moderate amount	Reference	Reference	Reference		Reference	Reference		Reference	Reference		Reference
	A lot or very much	1.92 (0.24-15.19)	.54	1.67 (0.86-3.24)		.13	—		—	—		—
**Frequency of** **Care Information Exchange** **use**												
	Once a month or less	Reference	Reference	Reference		Reference	Reference		Reference	Reference		Reference
	Once a week or more	1.05 (0.32-3.45)	.94	2.40 (1.59-3.63)		<.001	0.92 (0.24-3.60)		.90	2.31 (1.49-3.58)		<.001

^a^Goodness-of-fit: *χ*^2^_8_=14.5; *P*=.07.

^b^N/A: not applicable; variable not entered into the multivariable analyses due to nonsignificance (*P*>.25) in univariate analyses.

^c^Variable excluded from the final multivariable model using a backward elimination approach.

#### Frequency of CIE Use

Patients using CIE at least once per week were more likely to perceive improved care quality compared with patients using CIE less frequently (OR 2.31, 95% CI 1.49-3.58; *P*<.001). Sensitivity analyses assessing the effects of including or excluding predictor variables that had demonstrated significance in univariate analyses did not alter the results of the multivariable regression.

For the survey item “How has CIE changed how satisfied you are with your care?” the final multivariable model with 3 predictor variables (ethnicity, digital health literacy, and frequency of CIE use) predicted significantly better than the null (intercept) model (*P*<.001) and Pearson chi-square statistic suggested that the model fit the data well (*χ*^2^_8_=5.6; *P*=.69). Patients with higher digital health literacy (eHEALS score≥26) were more likely to report greater satisfaction with their care with CIE use, compared with those with lower digital health literacy (OR 2.35, 95% CI 1.09-5.04; *P*=.03; Table 4). CIE use was also associated with greater satisfaction with care among patients belonging to an ethnic minority group compared with White patients (OR 2.12, 95% CI 1.22-3.67; *P*=.007). Cross-tabulation of patients’ ethnicity and perceived change in satisfaction with care revealed that 77% (34/44) of Asian or British Asian patients reported greater satisfaction with care with CIE use, compared with 55% (11/20) of Black or African or Caribbean or Black British patients, 36% (4/11) of patients from mixed or multiple ethnic groups, and 42.1% (137/325; missing data n=18) of White patients (Table S2 in Multimedia Appendix 4).

Patients using CIE at least once per week were more likely to report greater satisfaction with care compared with patients using CIE less frequently (OR 2.03, 95% CI 1.31-3.14; *P*=.002).

Sensitivity analyses assessing the effects of including or excluding predictor variables that had demonstrated significance in univariate analyses did not alter the results of the multivariable analyses.

**Table 4:** Multinomial regression results of patients’ sociodemographic characteristics and impact of Care Information Exchange on patient’s satisfaction with care.

		Univariate^a^						Multivariable^a^				
		Worse care quality vs about the same			Better care quality vs about the same			Worse care quality vs about the same			Better care quality vs about the same	
		Odds ratio (95% CI)	*P* value	Odds ratio (95% CI)		*P* value	Odds ratio (95% CI)		*P* value	Odds ratio (95% CI)		*P* value
**Sex**												
	Female	Reference	Reference	Reference		Reference	Reference		Reference	Reference		Reference
	Male	0.84 (0.31-2.31)	.74	1.32 (0.88-1.97)		.17	—^b^		—	—		—
**Age (years)**												
	≥65	Reference	Reference	Reference		Reference	Reference		Reference	Reference		Reference
	≤64	0.69 (0.27-1.80)	.45	1.215 (0.76-1.75)		.51	N/A^c^		N/A	N/A		N/A
**Ethnicity**												
	White	Reference	Reference	Reference		Reference	Reference		Reference	Reference		Reference
	Ethnic minority	1.30 (0.35-4.78)	.70	2.32 (1.38-3.90)		.002	1.68 (0.44-6.41)		.45	2.12 (1.22-3.67)		.007
**Native language**												
	English	Reference	Reference	Reference		Reference	Reference		Reference	Reference		Reference
	Non-English	1.74 (0.47-6.52)	.41	1.63 (0.91-2.89)		.10	—		—	—		—
**Education**												
	Secondary or below	Reference	Reference	Reference		Reference	Reference		Reference	Reference		Reference
	Undergraduate or Professional	8.15 (1.03-64.80)	.05	1.14 (0.67-1.96)		.63	—		—	—		—
	Postgraduate or higher	5.94 (0.67-52.47)	.11	1.11 (0.69-1.80)		.67	—		—	—		—
**Digital literacy**												
	Lower digital health literacy	Reference	Reference	Reference		Reference	Reference		Reference	Reference		Reference
	Higher digital health literacy	2.29 (0.29-18.03)	.43	2.47 (1.19-5.13)		.02	2.17 (0.27-17.35)		.46	2.35 (1.09-5.04)		.03
**Health status**												
	Neither good nor poor	Reference	Reference	Reference		Reference	Reference		Reference	Reference		Reference
	Poor	0.92 (0.27-3.16)	.89	1.34 (0.80-2.23)		.27	N/A		N/A	N/A		N/A
	Good	1.04 (0.32-3.33)	.95	1.07 (0.65-1.78)		.78	N/A		N/A	N/A		N/A
**Motivation to be involved in own care**												
	Not very much or a moderate amount	Reference	Reference	Reference		Reference	Reference		Reference	Reference		Reference
	A lot or very much	3.00 (0.39-23.31)	.29	1.99 (1.04-3.82)		.04	X^d^		X	X		X
**Frequency of CIE use**												
	Once a month or less	Reference	Reference	Reference		Reference	Reference		Reference	Reference		Reference
	Once a week or more	0.92 (0.32-2.67)	.88	2.13 (1.41-3.23)		<.001	0.90 (0.27-2.95)		.86	2.03 (1.31-3.13)		.002

^a^Goodness-of-fit: *χ*^2^_8_=5.6; *P*=.69.

^b^Variable excluded from the final multivariable model using a backward elimination approach.

^c^N/A: not applicable; variable not entered into the multivariable analyses due to nonsignificance (*P*>.25) in univariate analyses.

^d^Variable excluded from the final multivariable model due to 0 cell counts producing unstable estimates.

### Missing Data Analysis

Of 523 survey respondents, 78 (14.9%), who had previously used CIE, had missing data regarding age and gender, and these respondents were excluded from our analyses. Meaningful comparisons of sociodemographic characteristics between the missing data sample and the analysis sample were not possible due to considerable additional missing data in the group of 78 respondents excluded from this analysis (Multimedia Appendix 5). There were no differences in the distribution of responses between the analysis sample and the missing data sample for the questionnaire item “How has CIE changed how satisfied you are with your care?” However, patients included in the analysis were more likely to view the impact of CIE on overall quality of care favorably, compared with those in the missing data sample (*χ*^2^_4_=10.3; *P*=.04; Multimedia Appendix 6).

## Discussion

### Principal Findings

Although many portal users perceived no change with CIE use, a large proportion reported better care quality and greater satisfaction with their care. Around 30% patients perceived their care to be safer, more effective, and more efficient with CIE, and approximately 40% reported that the timeliness of appointments, diagnoses, and treatments had improved. The most positive patient-perceived changes were in the domain of patient centeredness: more than half of patients using CIE felt more in control of their health care and able to play a greater role in decision-making. Patients from ethnic minority groups, those with higher digital health literacy, and those using CIE more frequently were more likely to perceive improvements in overall care quality and greater satisfaction with care. Across ethnic groups, patients of Asian or British Asian ethnicity reported the greatest benefits of portal use in terms of improving care quality and satisfaction with care received.

### Comparison With Wider Literature

These reports from users of a web-based patient portal in the United Kingdom are consistent with the findings of other patient experience studies in finding that many patients perceive a range of benefits associated with portal use [28-37]. To our knowledge, this is the first empirical study to map patients’ experiences against the 6 domains of quality of care to provide broad insight into the perceived effects of portal use from the patient perspective.

Regarding the domain of effectiveness, around 1 in 4 patients in our study believed that CIE use contributed to improving their overall health, and this finding echoes the results of other survey studies and meta-analyses of randomized trials [3,38]. We did not collect information about respondents’ medical histories; however, prior studies have shown that portal use may be particularly effective in supporting people with long-term conditions to improve their health, including those with diabetes and hypertension [3,38].

Existing evidence links patient portals to increased medication safety through patients possessing greater knowledge about their medicines, improved medication adherence, and increased reporting of medicine discrepancies [3,39-41]. Our study has shown that patients perceive additional safety impacts of web-based portals including improved accuracy of personal health information and detection of health record errors. Our previous work, together with studies conducted in the United States, has demonstrated that around 1 in 5 patients who access their web-based personal health records can, and do, notice errors in their records, and most patients would like to play an active role in rectifying these discrepancies [16,42]. Moreover, Blease et al [40] have shown that enabling informal carers to access the electronic health records of vulnerable patients (eg, people with serious mental illness) can help to prevent medication errors, delayed diagnoses, and other patient safety risks.

Regarding the efficiency domain, more than one-third of patients in our study perceived their own workload relating to their health had changed for the better. In a previous survey study in Canada, patients reported that web-based portals save time when scheduling appointments, patients needed to repeat themselves less during appointments, and portal use meant that patients could avoid unnecessary clinic visits [43]. Similarly, a review of randomized trials found a reduction in health care use (or no change) when patients have access to their electronic health records [3]. No experimental trials have investigated the impact of web-based portals on the timeliness of care delivery [3]; however, approximately 40% of the patients in our study perceived that CIE enabled them to access appointments, diagnoses, and treatment more quickly.

A growing body of evidence suggests that patients who are engaged in their care are more likely to adhere to medication and treatment plans, take up screening opportunities and prevention practices, participate in the detection of errors and safety risks, and adopt effective management strategies for chronic conditions [28,44-47]. The findings of this and numerous other survey studies have consistently found that patients feel more in control of their health care and better able to play a role in decision-making with access to their personal health records [28,33,34,37,40].

Regarding equity, our findings are consistent with previous research demonstrating that patients experiencing barriers to accessing web-based portals (including low digital literacy), and those with low levels of engagement in technology-enabled care are less likely to report that portals improve their health [38,48]. Previous research has also demonstrated that portal uptake is lower among patients belonging to ethnic minority groups [38]. However, in line with survey studies of portal users in the United States [28,29], we found that CIE users self-identifying to an ethnic minority group were more likely to report better care quality and greater satisfaction with care. Gerard et al [29] found that, compared with White patients, patients of Asian ethnicity in the United States were twice as likely to report the benefits of portal use; our study echoes this finding in the United Kingdom. Sharing electronic health records with patients appears to increase transparency and trust and strengthens the relationship between patients and their providers [44]. These benefits may be particularly important for ethnic minority groups to feel satisfied with their care; however, further qualitative research is needed to understand the mechanisms of portal adoption across different ethnic minority groups.

Of note, we found that patients who use CIE frequently were more likely to perceive improvements in overall care quality (and greater satisfaction with care). However, the direction of this effect is unclear. We suggest that this mechanism is likely to be circular, with initial portal use leading to perceived improvements in care quality, resulting in greater satisfaction with care, prompting increased portal use. In this way, the perception of quality of care could serve as a mechanism of sustained portal adoption. This theory is consistent with the Technology Acceptance Model, which suggests that use behavior (actual use) is partly predicated by the perceived benefits of using the technology [49]. In the study by Portz et al [50], which used the Technology Acceptance Model to explore portal use among older adults with chronic conditions, patient-perceived usefulness (communicating with care provider, saving time and money, addressing concerns without a clinic visit) was linked to frequent use of specific portal features, including the message center, pharmacy center, and viewing laboratory results. Further evaluation of CIE should include developing and testing a “Theory of Change” to determine how and why portal use leads to greater satisfaction with care in some patient groups [51].

### Policy Implications and Future Research

This study confirms the importance of addressing “the digital divide” as a policy priority to ensure equitable access to the benefits of patient portals for all patients [14,52]. Crosscutting interventions with system impacts, including user-centric design of portal platforms that adhere to accessibility, legibility and readability standards, and a commitment to “safety net” strategies such as the provision of low-cost, Wi-Fi–enabled devices or patient outreach programs, could all help to ensure that traditionally underserved groups can benefit from portal use [40,53]. More work is required to understand the relative effectiveness of these interventions, such that equity of access and adoption can be achieved for all patients. However, beyond literacy and technology access, our findings suggest that there are other potential avenues for addressing health disparities by expanding patient portal use to underrepresented groups. That ethnic minority groups see greater benefits in accessing their personal health records is worthy of further careful inquiry. Further research using qualitative methods would help to elucidate the mechanisms of patient portal adoption among ethnic minority communities.

### Strengths and Limitations

We mapped survey items to the Institute of Medicine’s 6 domains to provide a broad overview of perceptions of care quality among CIE users. However, our questionnaire was not designed to evaluate the domains of care quality as multidimensional constructs. There is a need to develop instruments that can measure subjective accounts of care quality as seen through the patient lens; developing and validating such a questionnaire could be the focus of future work.

We recruited a diverse sample, with one-third of respondents residing outside London and 1 in 5 self-identifying to an ethnic minority group. However, the numbers of patients in subgroups of ethnic minority were small. As such, we combined categories of ethnicity for the multivariable regression. Research exploring issues of equity should disaggregate ethnic categories where possible so the experiences of different ethnic groups can be understood [54]. Although we ran cross-tabulations to explore differences between ethnic groups, the numbers were small and may not generalize to larger populations.

Our web-based recruitment strategy may have introduced selection bias because web-based survey studies may favor the inclusion of patients who are digitally literate and more able to fully engage with patient portals. Our sample only included users of a web-based portal, and our findings are based on patient self-reported and perceived changes in care quality based on portal use. As such, and due to the nature of the study design, we cannot make any causal claims about the impact of patient portals on the quality of care. Building on limited existing evidence from controlled trials [2,3], further experimental or quasi-experimental studies should test the relationship between patient portal use and care quality using validated end points.

### Conclusions

A large proportion of CIE users perceived better overall quality of care and greater satisfaction with care, although many portal users reported no change. Perceived improvements were reported across all 6 domains of care quality, with the most favorable in the domain of patient centeredness. Patients from ethnic minority backgrounds (particularly Asian or British Asian) and those with higher digital health literacy perceived the greatest benefits of CIE use. With national policy directed toward addressing health disparities, patient portals could be valuable in improving care quality for patients in underrepresented groups, providing the needs of digitally disempowered patients are addressed. Further research should test the relationship between patient portal use and validated measures of the domains of care quality.

## Appendix

Multimedia Appendix 1 Cross-tabulation of patients’ sociodemographic characteristics and their perceptions of the impact of Care Information Exchange on satisfaction with care.

Multimedia Appendix 2 The Strengthening the Reporting of Observational Studies in Epidemiology Statement—Checklist of Items That Should Be Addressed in Reports of Observational Studies.

Multimedia Appendix 3 Patients’ perceptions of the impact of Care Information Exchange on (1) overall quality of care and (2) satisfaction with care.

Multimedia Appendix 4 Cross-tabulation of patients’ ethnicity and perceived change in overall quality of care with Care Information Exchange use.

Multimedia Appendix 5 Sociodemographic characteristics of patients in the missing data sample and in the analysis sample.

Multimedia Appendix 6 Missing data analysis for questionnaire items.

## Abbreviations

CIE: Care Information Exchange
eHEALS: eHealth Literacy Scale
OR: odds ratio
