# Supplementary material for: Perceptions of Quality of Care Among Users of a Web-Based Patient Portal: Cross-sectional Survey Analysis
Source: J Med Internet Res. 2022 Nov 17;24(11):e39973. doi: 10.2196/39973 (PMC9716419; doi:10.2196/39973)
Supplement: Multimedia Appendix 1 [file jmir_v24i11e39973_app1.docx]

Multimedia Appendix 1

Table S1. Cross-tabulation of patients’ sociodemographic characteristics and their perceptions of the impact of CIE on overall quality of care.

Survey item: Has CIE changed overall quality of care received?

|  |  | Much worse | Somewhat worse | About the same | Somewhat better | Much better |  |  |
| --- | --- | --- | --- | --- | --- | --- | --- | --- |
|  |  | n (%) | n (%) | n (%) | n (%) | n (%) | χ^2^ | *P* |
| Sex |  |  |  |  |  |  | 3.62 | .46 |
|  | Male | 2  (1.2) | 1  (0.6) | 89  (54.3) | 37  (22.6) | 35  (21.3) |  |  |
|  | Female | 5  (1.9) | 6  (2.3) | 154  (58.3) | 55  (20.8) | 44  (16.7) |  |  |
| Age |  |  |  |  |  |  | 12.32 | .72 |
|  | ≤30 | 1  (4.5) | 0  (0.0) | 9  (40.9) | 7  (31.8) | 5  (22.7) |  |  |
|  | 31-40 | 1  (2.1) | 2  (4.2) | 24  (50.0) | 13  (27.1) | 8  (16.7) |  |  |
|  | 41-50 | 1  (1.7) | 2  (3.4) | 32  (55.2) | 9  (15.5) | 14  (24.1) |  |  |
|  | 51-64 | 2  (1.2) | 1  (0.6) | 93  (57.8) | 35  (21.7) | 30  (18.6) |  |  |
|  | ≥65 | 2  (1.4) | 2  (1.4) | 85  (60.7) | 29  (20.7) | 22  (15.7) |  |  |
| Ethnicity |  |  |  |  |  |  | 15.17 | .004 |
|  | Ethnic minority | 0  (0.0) | 3  (3.8) | 32  (40.5) | 21  (26.6) | 23  (29.1) |  |  |
|  | White | 6  (1.8) | 4  (1.2) | 200  (60.4) | 67  (20.2) | 54  (16.3) |  |  |
|  |  |  |  |  |  |  |  |  |
| Education |  |  |  |  |  |  | 6.01 | .65 |
|  | Secondary school or below | 1  (0.9) | 0  (0.0) | 65  (55.6) | 30  (25.6) | 21  (17.9) |  |  |
|  | Undergraduate/ professional degree | 4  (2.3) | 3  (1.7) | 99  (57.6) | 38  (22.1) | 28  (16.3) |  |  |
|  | Postgraduate or higher | 2  (1.9) | 2  (1.9) | 65  (61.3) | 17  (16.0) | 20  (18.9) |  |  |
| Language |  |  |  |  |  |  | 5.62 | .23 |
|  | Non-english | 1  (1.7) | 2  (3.4) | 25  (43.1) | 17  (29.3) | 13  (22.4) |  |  |
|  | English | 5  (1.4) | 5  (1.4) | 213  (58.5) | 76  (20.9) | 65  (17.9) |  |  |
| Digital Literacy |  |  |  |  |  |  | 13.15 | .01 |
|  | High digital literacy | 3  (0.9) | 6  (1.9) | 165  (52.1) | 77  (24.3) | 66  (20.8) |  |  |
|  | Low digital literacy | 2  (2.1) | 1  (1.1) | 67  (71.3) | 15  (16.0) | 9  (9.6) |  |  |
| Health status |  |  |  |  |  |  | 8.63 | .37 |
|  | Good | 2  (1.2) | 2  (1.2) | 95  (56.5) | 33  (19.6) | 36  (21.4) |  |  |
|  | Neutral | 4  (3.8) | 1  (1.0) | 62  (59.6) | 23  (22.1) | 14  (13.5) |  |  |
|  | Poor | 1  (0.6) | 4  (2.5) | 86  (54.8) | 37  (23.6) | 29  (18.5) |  |  |
| Motivation to be involved in own care |  |  |  |  |  |  | 11.07 | .52 |
|  | Very much | 4  (1.5) | 6  (2.3) | 137  (51.5) | 60  (22.6) | 59  (22.2) |  |  |
|  | A lot | 2  (1.7) | 1  (0.9) | 73  (63.5) | 25  (21.7) | 14  (12.2) |  |  |
|  | A moderate amount | 1  (2.4) | 0  (0.0) | 28  (68.3) | 7  (17.1) | 5  (12.2) |  |  |
|  | Not very much | 0  (0.0) | 0  (0.0) | 3  (60.0) | 1  (20.0) | 1  (20.0) |  |  |
|  |  |  |  |  |  |  |  |  |

Table S2. Cross-tabulation of patients’ sociodemographic characteristics and their perceptions of the impact of CIE on satisfaction with care.

Survey item: Has CIE changed satisfaction with care received?

|  |  | Much worse | Somewhat worse | About the same | Somewhat better | Much better |  |  |
| --- | --- | --- | --- | --- | --- | --- | --- | --- |
|  |  | n (%) | n (%) | n (%) | n (%) | n (%) | χ^2^ | *P* |
| Sex |  |  |  |  |  |  | 2.53 | .64 |
|  | Male | 2  (1.2) | 4  (2.5) | 75  (46.3) | 42  (25.9) | 39  (24.1) |  |  |
|  | Female | 5  (1.9) | 8  (3.1) | 137  (52.3) | 54  (20.6) | 58  (22.1) |  |  |
| Age |  |  |  |  |  |  | 12.90 | .68 |
|  | ≤30 | 1  (4.8) | 0  (0.0) | 6  (28.6) | 8  (38.1) | 6  (28.6) |  |  |
|  | 31-40 | 1  (2.1) | 2  (4.2) | 22  (45.8) | 14  (29.2) | 9  (18.8) |  |  |
|  | 41-50 | 0  (0.0) | 2  (3.4) | 27  (45.8) | 16  (27.1) | 14  (23.7) |  |  |
|  | 51-64 | 2  (1.3) | 3  (1.9) | 86  (54.1) | 30  (18.9) | 38  (23.9) |  |  |
|  | ≥65 | 3  (2.2) | 5  (3.6) | 71  (51.4) | 29  (21.0) | 30  (21.7) |  |  |
| Ethnicity |  |  |  |  |  |  | 12.63 | .01 |
|  | Ethnic minority | 0  (0.0) | 3  (3.8) | 27  (34.2) | 22  (27.8) | 27  (34.2) |  |  |
|  | White | 6  (1.8) | 9  (2.8) | 175  (53.5) | 69  (21.1) | 68  (20.8) |  |  |
|  |  |  |  |  |  |  |  |  |
| Education |  |  |  |  |  |  | 9.42 | .31 |
|  | Secondary school or below | 1  (0.9) | 0  (0.0) | 63  (55.3) | 20  (17.5) | 30  (26.3) |  |  |
|  | Undergraduate/ professional degree | 4  (2.3) | 7  (4.1) | 85  (49.7) | 43  (25.1) | 32  (18.7) |  |  |
|  | Postgraduate or higher | 2  (1.9) | 3  (2.8) | 53  (50.0) | 24  (22.6) | 24  (22.6) |  |  |
| Language |  |  |  |  |  |  | 3.67 | .45 |
|  | Non-english | 1  (1.7) | 2  (3.4) | 23  (39.7) | 18  (31.0) | 14  (24.1) |  |  |
|  | English | 5  (1.4) | 9  (2.5) | 187  (51.8) | 78  (21.6) | 82  (22.7) |  |  |
| Digital Literacy |  |  |  |  |  |  | 12.92 | .01 |
|  | High digital literacy | 4  (1.3) | 8  (2.5) | 149  (47.3) | 68  (21.6) | 86  (27.3) |  |  |
|  | Low digital literacy | 1  (1.1) | 3  (3.2) | 55  (58.2) | 26  (27.7) | 9  (9.6) |  |  |
| Health status |  |  |  |  |  |  | 16.16 | .04 |
|  | Good | 3  (1.8) | 5  (3.0) | 85  (51.2) | 29  (17.5) | 44  (26.5) |  |  |
|  | Neutral | 4  (3.8) | 1  (1.0) | 55  (52.9) | 29  (27.9) | 15  (14.4) |  |  |
|  | Poor | 0  (0.0) | 6  (3.9) | 72  (46.5) | 39  (25.2) | 38  (24.5) |  |  |
| Motivation to be involved in own care |  |  |  |  |  |  | 20.01 | .07 |
|  | Very much | 3  (1.1) | 8  (3.0) | 120  (45.6) | 57  (21.7) | 75  (28.5) |  |  |
|  | A lot | 3  (2.6) | 4  (3.5) | 60  (52.6) | 32  (28.1) | 15  (13.2) |  |  |
|  | A moderate amount | 1  (2.4) | 0  (0.0) | 28  (68.3) | 7  (17.1) | 5  (12.2) |  |  |
|  | Not very much | 0  (0.0) | 0  (0.0) | 2  (40.0) | 1  (20.0) | 2  (20.0) |  |  |
|  |  |  |  |  |  |  |  |  |
