# Supplementary material for: Perceptions of Quality of Care Among Users of a Web-Based Patient Portal: Cross-sectional Survey Analysis
Source: J Med Internet Res. 2022 Nov 17;24(11):e39973. doi: 10.2196/39973 (PMC9716419; doi:10.2196/39973)
Supplement: Multimedia Appendix 2 [file jmir_v24i11e39973_app2.docx]

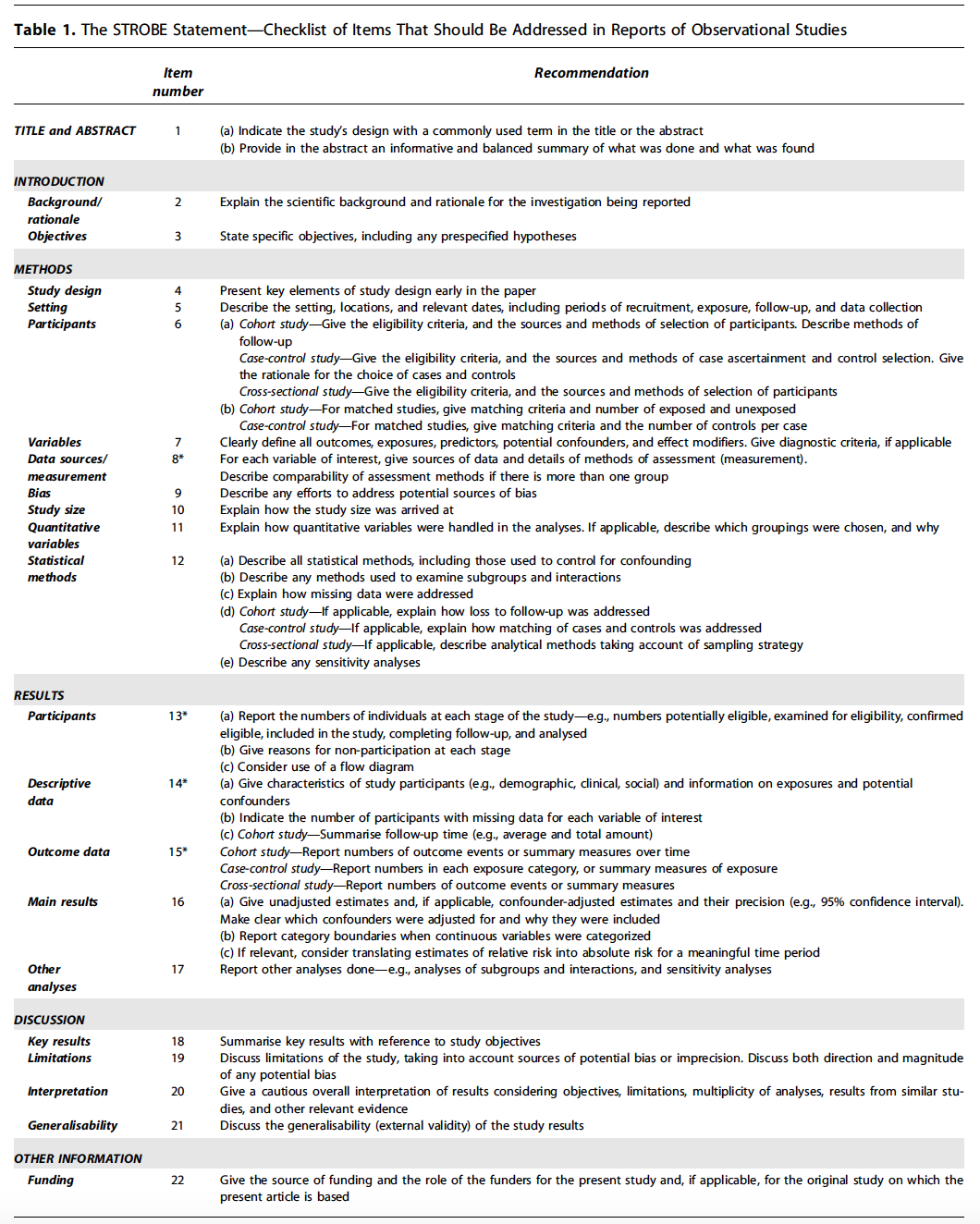
Multimedia Appendix 2

Source:

Vandenbroucke JP, Von Elm E, Altman DG, Gøtzsche PC, Mulrow CD, Pocock SJ, STROBE Initiative. Strengthening the reporting of observational studies in epidemiology (STROBE): explanation and elaboration. PLoS Med 2007 Oct 16;4(10):e297
