# Supplementary material for: Perceptions of Quality of Care Among Users of a Web-Based Patient Portal: Cross-sectional Survey Analysis
Source: J Med Internet Res. 2022 Nov 17;24(11):e39973. doi: 10.2196/39973 (PMC9716419; doi:10.2196/39973)
Supplement: Multimedia Appendix 3 [file jmir_v24i11e39973_app3.docx]

Multimedia Appendix 3

Table S1. Patients’ perceptions of the impact of CIE on i) overall quality of care and ii) satisfaction with care

|  | Missing data  n (%) | Much worse  n (%) | Somewhat worse  n (%) | About the same  n (%) | Somewhat better  n (%) | Much better  n (%) |
| --- | --- | --- | --- | --- | --- | --- |
|  |  |  |  |  |  |  |
| How has CIE changed the overall quality of care you have received? | 16  (3.6) | 7  (1.6) | 7  (1.6) | 243  (54.6) | 93  (20.9) | 79  (17.8) |
| How has CIE changed how satisfied you are with your care? | 20  (4.5) | 7  (1.6) | 12  (2.7) | 212  (47.6) | 97  (21.8) | 97  (21.8) |
