# Supplementary material for: Perceptions of Quality of Care Among Users of a Web-Based Patient Portal: Cross-sectional Survey Analysis
Source: J Med Internet Res. 2022 Nov 17;24(11):e39973. doi: 10.2196/39973 (PMC9716419; doi:10.2196/39973)
Supplement: Multimedia Appendix 4 [file jmir_v24i11e39973_app4.docx]

Multimedia Appendix 4

Table S1. Cross-tabulation of patients’ ethnicity and perceived change in overall quality of care with CIE use.

Survey item: Has CIE changed overall quality of care received?

|  | Much worse  n (%) | Somewhat worse  n (%) | About the same  n (%) | Somewhat better  n (%) | Much better  n (%) |
| --- | --- | --- | --- | --- | --- |
|  |  |  |  |  |  |
| Asian/British Asian | 0  (0.0) | 0  (0.0) | 14  (31.8) | 14  (31.8) | 16  (36.4) |
| Black/African/Caribbean/ Black British | 0  (0.0) | 1  (5.0) | 10  (50.0) | 4  (20.0) | 5  (25.0) |
| Mixed/multiple ethnic groups | 0 (0.0) | 2  (18.2) | 5  (45.5) | 2  (18.2) | 2  (18.2) |
| Other | 0  (0.0) | 0  (0.0) | 14  (66.7) | 4  (19.0) | 3  (14.3) |
| White | 6  (1.8) | 4  (1.2) | 198  (60.4) | 67  (20.4) | 53  (16.2) |
| No response for ethnicity | 1  (20.0) | 0  (0.0) | 2  (40.0) | 2  (40.0) | 0  (0.0) |

Table S2. Cross-tabulation of patients’ ethnicity and perceived change in satisfaction with care with CIE use.

Survey item: Has CIE changed satisfaction with care received?

|  | Much worse  n (%) | Somewhat worse  n (%) | About the same  n (%) | Somewhat better  n (%) | Much better  n (%) |
| --- | --- | --- | --- | --- | --- |
|  |  |  |  |  |  |
| Asian/British Asian | 0  (0.0) | 0  (0.0) | 10  (22.7) | 15  (34.1) | 19  (43.2) |
| Black/African/Caribbean/ Black British | 0  (0.0) | 1  (5.0) | 8  (40.0) | 4  (20.0) | 7  (35.0) |
| Mixed/multiple ethnic groups | 0 (0.0) | 2  (18.2) | 5  (45.5) | 3  (27.3) | 1  (9.1) |
| Other | 0  (0.0) | 0  (0.0) | 14  (70.0) | 4  (20.0) | 2  (10.0) |
| White | 6  (1.8) | 9  (2.8) | 173  (53.2) | 69  (21.2) | 68  (20.9) |
| No response for ethnicity | 1  (20.0) | 0  (0.0) | 2  (40.0) | 2  (40.0) | 0  (0.0) |
