# Supplementary material for: Perceptions of Quality of Care Among Users of a Web-Based Patient Portal: Cross-sectional Survey Analysis
Source: J Med Internet Res. 2022 Nov 17;24(11):e39973. doi: 10.2196/39973 (PMC9716419; doi:10.2196/39973)
Supplement: Multimedia Appendix 6 [file jmir_v24i11e39973_app6.docx]

Multimedia Appendix 6

Table S1: Missing data analysis for questionnaire items

|  | Missing data^(a)^  sample | Included sample |  |  |
| --- | --- | --- | --- | --- |
|  | n (%) | n (%) | χ^2^_4_ | *P* |
| Has CIE changed satisfaction with care? | | | 5.65 | .28 |
| Much worse | 3 (6.8) | 7 (1.6) |  |  |
| Somewhat worse | 2 (4.5) | 12 (2.8) |  |  |
| About the same | 20 (45.5) | 212 (49.9) |  |  |
| Somewhat better | 9 (20.5) | 97 (22.8) |  |  |
| Much better | 10 (22.7) | 97 (22.8) |  |  |
| Has CIE changed overall quality of care received? | | | 10.28 | .04 |
| Much worse | 2 (4.2) | 7 (1.6) |  |  |
| Somewhat worse | 4 (8.3) | 7 (1.6) |  |  |
| About the same | 25 (52.1) | 243 (56.6) |  |  |
| Somewhat better | 9 (18.8) | 93 (21.7) |  |  |
| Much better | 8 (16.7) | 79 (18.4) |  |  |

^(a)^Respondents who did not provide basic characterization regarding age and sex.
